# An Analysis of the Comparative Efficacy Between a Third-Generation and a Second-Generation Supraglottic Airway Device in Patients Undergoing Laparoscopic Cholecystectomy

**DOI:** 10.7759/cureus.22592

**Published:** 2022-02-25

**Authors:** Darvish Hussain, Raksha Kundal, Anil Kumar, Nikki Sabharwal

**Affiliations:** 1 Anaesthesiology, Lady Hardinge Medical College, New Delhi, IND; 2 Anaesthesiology, Vardhman Mahavir Medical College and Safdarjung Hospital, New Delhi, IND

**Keywords:** laryngopharyngeal morbidity, laparoscopic cholecystectomy, ease of insertion, oropharyngeal seal pressure, proseal lma, baska mask

## Abstract

Background and objective

Supraglottic airway devices are extensively used nowadays to secure the airway and minimize postoperative airway-related complications. This study aimed to evaluate whether the Baska^®^ mask (BM) provides higher seal pressure and a better first-time insertion compared to the laryngeal mask airway (LMA) ProSeal™ (LMA-P) in adult laparoscopic cholecystectomy.

Methodology

This prospective, randomized, single-blinded interventional study was performed after obtaining ethical approval from the Institutional Ethics Committee at the Vardhman Mahavir Medical College and Safdarjung Hospital, New Delhi. Sixty adult patients of both genders scheduled for laparoscopic cholecystectomy under general anesthesia were divided into two groups, with 30 patients in each group. Our study observed the number of insertion attempts, time of insertion, oropharyngeal seal pressure (OSP), number of patients requiring manipulation for proper placement of supraglottic airway devices, and ease of insertion.

Results

There were no significant differences in terms of insertion attempts, ease of insertion, and laryngopharyngeal morbidity between the groups. The mean OSP at five minutes was 31.55 ±2.23 cm H_2_O, and that at 30 minutes was 35.86 ±3.70 cm H_2_0 in the BM group, while in the LMA-P group, it was 24.17 ±3.74 cm H_2_0 and 25.97 ±3.79 cm H_2_0 respectively (p<0.001). In our study, the trend of OSP continued to increase in the BM group more than in the LMA-P group during surgery.

Conclusion

The BM provided better OSP than the LMA-P, which was observed throughout the surgery.

## Introduction

Supraglottic airway devices have been used extensively to manage various laparoscopic surgeries [1]. Adequate (mean value: 19.5-21.3) oropharyngeal seal pressure (OSP) is the key to maintaining optimal ventilation and airway protection from aspiration [2]. The recently developed Baska® mask (BM) is a single-use device with a non-inflatable cuff made of silicone [3]. This device takes the shape of a supraglottic airway and inflates and deflates with positive pressure ventilation, thereby preventing leaks. In addition, it contains side channels for suctioning aspirates, oesophageal drains, and an integrated bite block. It also has a second oropharyngeal curve that can be manipulated by pulling the tab of the BM. The second-generation laryngeal mask airway (LMA) has been designed to provide a higher airway seal and is relatively safer because it has a gastric channel [4].

There is scant data in the literature regarding the comparison between these two devices in adult laparoscopic cholecystectomy. In light of this, we conducted this study with the primary aim of comparatively evaluating the seal pressure of the BM with that of the LMA ProSeal™ (LMA-P). Secondary objectives were the comparative evaluation of ease of insertion, the time required for successful placement, percentage of successful first attempts, number of attempts required for insertion, and manipulation, if any, needed for the placement of these two devices. We hypothesized that by virtue of having a non-inflatable cuff and insertion tab, the BM might have higher OSP and enables easier insertion than LMA-P in adult patients undergoing laparoscopic cholecystectomy.

## Materials and methods

**Figure 1:**
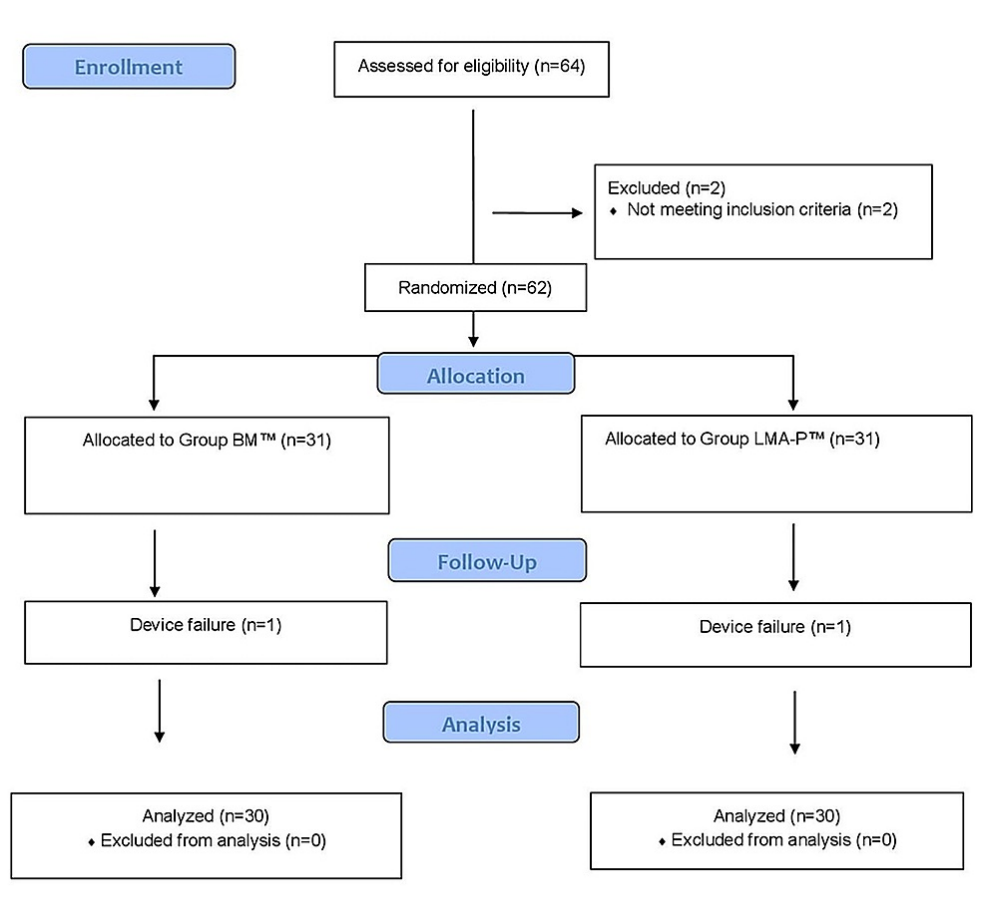
Consort flow diagram BM: Baska mask; LMA-P: laryngeal mask airway ProSeal

This prospective randomized controlled trial was conducted at a tertiary care center in India after gaining approval from the hospital ethics committee (IEC/VMMC/SJH/Thesis/Oct 2017-015). After obtaining written informed consent, we analyzed 60 patients (Figure 1; consort flow diagram) with the American Society of Anesthesiologists (ASA) physical status I or II who were aged 18-65 years and scheduled for elective laparoscopic cholecystectomy. We excluded patients with an anticipated difficult airway, high risk of aspiration (gastroesophageal reflux or treated disease), preoperative sore throat/upper respiratory tract infection, BMI >30 kg/m^2^, and those who refused to provide informed consent. The patients were divided into two groups: the BM group and the LMA-P group. Randomization was performed using a computer-generated random number table, and the devices were allocated using the sealed envelope technique. Although blinding was not possible during device insertion, it was maintained during data analysis.

On arrival in the operating room after being connected with standard monitors, each patient received general anesthesia with injection fentanyl 2 µg/kg body weight and propofol 2 mg/kg body weight and using vecuronium bromide as a muscle relaxant intravenously. Face mask ventilation was performed with O_2_, N_2_O (1:1), and isoflurane 0.8-1.2% for three minutes, and then an appropriate airway device as per the allocated group was inserted. All supraglottic device insertions were performed by anesthesiologists trained in the insertion of the BM and LMA-P in adult patients before the start of the study. The size of the devices was decided as per the patients' weight and manufacturers' recommendations.

The cuff of the LMA-P was fully deflated, the dorsal surface was lubricated with water-soluble jelly, and the device was inserted with the help of an introducer with the patient's head and neck in sniffing position [4]. After placement, the cuff of the LMA-P was inflated with air to 60 cm H_2_O and maintained at this pressure throughout anesthesia using a cuff pressure gauge (Mallinckrodt Laboratories, Neunkirchen-Seelscheid, Germany). Intracuff pressure monitoring was performed every 30 minutes and adjusted to 60 cm H_2_O. The BM was lubricated with water-soluble lubricating jelly on the ventral surface; A BM of appropriate size was inserted with the patient's head in a sniffing position.

Under adequate depth of anesthesia, the proximal firmer part of the mask was compressed between the thumb and two fingers, and the device was advanced towards the hard and soft palate till resistance was felt; gentle pulling of the tab on the device was used to increase the palatopharyngeal curve to assist insertion. The ease of insertion was graded according to the resistance felt during insertion - grade I: no resistance to insertion, grade II: slight resistance to insertion, grade III: moderate resistance to insertion, and grade IV: high resistance/impossible to insert.

The airway tube of the devices was connected to a closed circuit. The adequate airway placement of the LMA was said to be present if there was bilateral symmetrical chest expansion on manual ventilation, bilateral equal air entry on auscultation, and 6 square waveform tracings on the capnograph. Also, less than 20% loss of setting the tidal volume on a ventilator, lack of gastric insufflation, and a lack of audible leak at a peak airway pressure of 20 cm of water during manual ventilation were noted. Minor airway manipulations such as jaw thrust while inserting the device, head and neck flexion or extension, chin lift, and change in the depth of device needed for achieving adequate airway were noted. An optimal insertion of the device was deemed to be attained if both an effective airway and a successful gastric tube insertion were achieved. After two failed insertion attempts, the airway was secured with an appropriately sized endotracheal tube.

Anesthesia was maintained with N_2_O 67%, O_2_ 33%, with isoflurane 0.6-0.8%, targeting end-tidal carbon dioxide (EtCO_2_) of 35-45 mmHg and end-tidal minimum alveolar concentration of 1.0-1.2. Supplementation of vecuronium bromide 1 mg intravenously was given as per requirement. Intramuscular diclofenac at a dose of 1.5 mg/kg body weight was given for pain relief, and injection ondansetron 75 µg/kg body weight intravenously was given as an antiemetic before the end of surgery. Ventilatory parameters like inspiratory tidal volume (ITV), expiratory tidal volume (ETV), EtCO_2_, peak airway pressure, and intrabdominal pressure were noted at one minute, five minutes, and 30 minutes after pneumoperitoneum and throughout the surgery. We calculated the leak fraction, i.e., ITV-ETV/ITV multiplied by 100 at five minutes post device insertion after connecting the patient to the ventilator.

OSP was measured after one minute and five minutes after pneumoperitoneum, and 30 minutes after connecting to a ventilator. We measured OSP by closing the circle system's expiratory valve at the fixed gas flow of 3 l/minute and noting the airway pressure (maximum of 40 cm H_2_O allowed) at which equilibrium was reached. Audible air leak near thyroid cartilage was assessed by auscultation, and the presence or absence of gastric insufflation by epigastric auscultation was also checked during leak pressure testing. We also recorded numbers of insertion attempts, time of insertion (holding the device at teeth level to obtain 4-6 square waveforms), the number of patients requiring manipulation or additional propofol for achieving effective airway, ease of insertion of the device (easy, slightly difficult, difficult, impossible).

At the end of the surgery, neuromuscular blockade was reversed with IV neostigmine and glycopyrrolate at a dose of 0.05 mg/kg and 0.01 mg/kg respectively. The device was removed in fully awake patients with their mouths open and suctioned continuously. We also assessed laryngopharyngeal morbidity by inspecting the device for blood stains or any sign of visible trauma to the patient's lips, tongue, teeth, or oral tissue, as this finding correlated with postoperative sore throat dysphagia and hoarseness.

Statistical analysis

Sample size calculation was based on the study by Alexiev et al. [5]. The observed mean sealing pressure was significantly higher in the BM group compared to the classic laryngeal mask airway group (29.98 ±8.51 vs. 24.50 ±6.19, p=0.013) [5]. Taking these values as a reference, the minimum required sample size with an 80% power of study and 5% level of significance was calculated to be 29 patients in each study group. Hence, we ultimately decided on a sample size of 60 (30 patients per group).

A total of 64 patients were enrolled by factoring in potential dropouts. Compiled data were tabulated and statistically analyzed using the SPSS Statistics software version 17.0 (IBM, Armonk, NY). Qualitative variables were expressed as frequencies/percentages and compared using the chi-squared/Mann-Whitney U test. Continuous variables were presented as mean ±SD. All quantitative variables were compared using the Student's t-test/Mann-Whitney U test. A p-value <0.05 was considered statistically significant.

## Results

Sixty-four patients were approached for enrollment in the study, out of which four patients were excluded. Finally, 60 participants were recruited for this randomized control trial that compared BM with LMA-P. One patient each in the BM group and LMA-P group was excluded from the study due to poor fit and excessive air leak. There was no statistical difference in terms of age, sex, and Mallampati scores between these groups (Table 1).

**Table 1:** Comparison of demographic data and supraglottic device sizes BM: Baska mask; LMA-P: laryngeal mask airway ProSeal; SD: standard deviation

Parameters	BM (n=30)	LMA-P (n=30)	P-value
Age, years, mean ±SD	32 ±13.67	35 ±14.38	0.576
Sex (female/male)	14/16	13/17	0.795
Mallampati score (I/II/III)	23/1/6	26/2/2	0.284
LMA size 3/4	13/17	9/21	0.272

We found a statistically significant difference in OSPs between the two devices (p<0.001), as shown in Table 2. Parameters regarding the performance of devices and their related complications are shown in Table 3.

**Table 2:** Comparison of OSP outcomes between the two groups BM: Baska mask; LMA-P: laryngeal mask airway ProSeal; OSP: oropharyngeal seal pressure; CTV: connection to ventilator; SD: standard deviation

Parameters	BM (n=30)	LMA-P (n=30)	P-value
OSP after 1 minute of CTV, mean ±SD	31.55 ±2.23 cm H_2_O	24.17 ±3.74 cm H_2_O	<0.001
OSP after 5 minutes of CTV, mean ±SD	31.66 ±2.45 cm H_2_0	24.33 ±3.66 cm H_2_O	<0.001
OSP after pneumoperitoneum, mean ±SD	33.31 ±2.45 cm H_2_0	25.97 ±3.79 cm H_2_O	<0.001
OSP after 30 minutes of CTV, mean ±SD	35.86 ±3.70 cm H_2_0	25.97 ±3.79 cm H_2_O	<0.001

**Table 3:** The performance of devices and related complications BM: Baska mask; LMA-P: laryngeal mask airway ProSeal; SD: standard deviation

Parameters	BM (n=30)	LMA-P (n=30)	P-value
Number of attempts (1/2)	24/6	28/2	0.254
Insertion time seconds, mean ±SD	25.33 ±3.62	28.77 ±6.6	0.004
Additional dose of propofol	3	0	0.237
Manipulation required	7	3	0.299
Postoperative complication after 1 hour			
Sore throat	1	3	0.612
Hoarseness	0	0	-
Dysphagia	0	0	-

The two groups were comparable in terms of the number of insertion attempts, additional doses of propofol needed, the requirement of device manipulation, and postoperative laryngopharyngeal morbidity. The insertion time for LMA-P was significantly longer than that for BM (p=0.004). Grade 1 ease of insertion in the BM group was observed in 23/30 cases (76.67%), while in the LMA-P group, it was present in 26/30 cases (86.67%), which was comparable between two devices (p=0.284).

The intraabdominal pressure, ITV, ETV, and EtCO2 values were comparable between the two groups in this study. The mean value for peak airway pressure after 30 minutes was 21.07 ±2.49 cm H_2_O in the BM group and 22.47 ±2.65 cm H_2_O in the LMA-P group; the peak airway pressure trend increased in both the groups. The difference in peak airway pressure after 30 minutes was statistically significant (p=0.016) (Table 4).

**Table 4:** Ventilatory parameters after connection to a ventilator (CTV) *Significant p-value BM: Baska mask; LMA-P: laryngeal mask airway ProSeal; CTV: connection to ventilator; SD: standard deviation

Parameters	BM (n=30), mean ±SD	LMA-P (n=30), mean ±SD	P-value
Intraabdominal pressure			
1 minute after CTV	0 ±0	0 ±0	-
5 minutes after CTV	0 ±0	0 ±0	-
After pneumoperitoneum	12 ±0	12 ±0	-
30 minutes after CTV	12 ±0	12 ±0	-
Inspiratory tidal volume			
1 minute after CTV	438.1 ±82.3 ml	438.1 ±82.3 ml	0.715
5 minutes after CTV	438.1 ±82.3 ml	438.1 ±82.3 ml	0.715
After pneumoperitoneum	438.1 ±82.3 ml	438.1 ±82.3 ml	0.715
30 minutes after CTV	438.1 ±82.3 ml		0.715
Expiratory tidal volume			
1 minute after CTV	423.28 ±79.91 ml	423.28 ±79.91 ml	0.996
5 minutes after CTV	427.48 ±81.94 ml	427.48 ±81.94 ml	0.752
After pneumoperitoneum	425.72 ±80.5 ml	422.63 ±57.64 ml	0.866
30 minutes after CTV	423.9 ±80.57 ml	421.63 ±57.34 ml	0.901
Peak airway pressure			
1 minute after CTV	19.52 ±2.05 cm H_2_0	20.3 ±2.22 cm H_2_0	0.079
5 minutes after CTV	19.52 ±2.05 cm H_2_0	20.5 ±2.37 cm H_2_0	0.064
After pneumoperitoneum	20.7 ±2.46 cm H_2_0	22.1 ±2.71 cm H_2_0	0.035
30 minutes after CTV	21.0 ±2.49 cm H_2_0	22.5 ±2.65 cm H_2_0	0.016*

In this study, the mean value for leak fraction at five minutes of connection to ventilator was 2.5 ±2.4% in the BM group and 3.49 ±3% in the LMA-P group. The difference in leak pressure between groups was not statistically significant (p=0.174).

## Discussion

Our study compared the BM with LMA-P in patients undergoing elective laparoscopic cholecystectomy. The principal finding of our study was a significant difference between OSP and insertion time of the BM as compared to LMA-P. Most of the measured variables like patient age, sex, Mallampati score, sizes of LMA, duration of surgery, and intraabdominal pressure during laparoscopic surgery were comparable in both groups. There was a significant difference between peak airway pressure after 30 minutes of connecting to the ventilator, but other ventilatory parameters were comparable.

In our study, both the devices were easy to insert; however, 23 (76.67%) patients in the BM group and 26 (86.67%) patients in the LMA-P group had grade 1 ease of insertion. The ease of insertion between the two groups was comparable with a p-value of 0.284. Our results were similar to the findings of Kachakayala et al. They also found the ease of insertion comparable between these two devices [6]. In the BM group, 23.33% of patients required manipulation, while 10% of patients required manipulation in the LMA-P group. This difference between the two groups was not statistically significant (p=0.299). The frequency of manipulation was relatively less in our study when compared to Alexiev et al., where additional maneuvers were required in 45% of the patients [5].

Our results suggested that the mean time for insertion in the BM group (25.33 ±3.62 seconds) was comparatively shorter than that in the LMA-P group (28.77 ±6.6 seconds) (p=0.004), which is very similar to the findings in other studies [5-10]. The reason behind the quick and easy insertion of BM may be attributed to its manufacturing design, which includes a cuffless membrane and a tab to increase the palatopharyngeal curve to assist insertion. However, Reddy et al. reported significantly increased insertion time for the BM group when compared to the LMA-P group [11].

Both BM and LMA-P are supraglottic airway devices with an in-situ gastric channel. It is well-known that LMA-P has higher seal pressures than i-gel, and BM also has higher seal pressure than i-gel [12,13]. The higher seal pressure of LMA-P may be due to the additional posterior cuff; however, BM lacks any such cuff. We observed higher OSP with BM compared to LMA-P. The mean OSP at different time intervals varied from 31.55 to 35.86 cm H_2_O in BM, while in the LMA-P group, it was 24.17 to 25.97 cm H_2_O (p<0.001). This finding was corroborated in the study by Singh et al., who observed OSP of 30.25 ±8.34 cm H_2_O for the BM and 23.50 ±4.05 cm H_2_O for the LMA-P [7]. Also, our findings are similar to those by Dhanasekaran et al. in 2019 and Ali et al. in 2013; both of them affirmed that the BM has significantly higher OSP than the LMA-P [8,10].

The OSP trend kept increasing in the BM group much more than the LMA-P group during surgery in our study, which is a new finding. This may be attributed to the BM having a membranous cuff, and its seal pressure directly correlating with the increase in intermittent positive pressure ventilation. It is a well-known fact that laparoscopic cholecystectomy requires higher intermittent positive pressure ventilation, including higher positive end-expiratory pressure [14]. There was no significant difference in laryngopharyngeal morbidity between the groups, which is in line with other studies [8-10].

Our study has some limitations. We did not include obese and pediatric patients due to the non-availability of suitable sizes of the BM. Moreover, this was a single-blinded study.

## Conclusions

Based on our findings, the BM provides higher OSP than the LMA-P and offers better protection against aspiration in laparoscopic cholecystectomy. Hence, we emphasize that the BM is a safer alternative than LMA-P in laparoscopic cholecystectomy.
